# Multiracial Facial Golden Ratio and Evaluation of Facial Appearance

**DOI:** 10.1371/journal.pone.0142914

**Published:** 2015-11-12

**Authors:** Mohammad Khursheed Alam, Nor Farid Mohd Noor, Rehana Basri, Tan Fo Yew, Tay Hui Wen

**Affiliations:** 1 Orthodontic Unit, School of Dental Science, Universiti Sains Malaysia, Kota Bharu, Kelantan, Malaysia; 2 Anatomy Department, School of Dental Science, Universiti Sains Malaysia, Kota Bharu, Kelantan, Malaysia; 3 Craniofacial Biology, School of Dental Science, Universiti Sains Malaysia, Kota Bharu, Kelantan, Malaysia; University of Tuebingen Medical School, GERMANY

## Abstract

This study aimed to investigate the association of facial proportion and its relation to the golden ratio with the evaluation of facial appearance among Malaysian population. This was a cross-sectional study with 286 randomly selected from Universiti Sains Malaysia (USM) Health Campus students (150 females and 136 males; 100 Malaysian Chinese, 100 Malaysian Malay and 86 Malaysian Indian), with the mean age of 21.54 ± 1.56 (Age range, 18–25). Facial indices obtained from direct facial measurements were used for the classification of facial shape into short, ideal and long. A validated structured questionnaire was used to assess subjects’ evaluation of their own facial appearance. The mean facial indices of Malaysian Indian (MI), Malaysian Chinese (MC) and Malaysian Malay (MM) were 1.59 ± 0.19, 1.57 ± 0.25 and 1.54 ± 0.23 respectively. Only MC showed significant sexual dimorphism in facial index (P = 0.047; P<0.05) but no significant difference was found between races. Out of the 286 subjects, 49 (17.1%) were of ideal facial shape, 156 (54.5%) short and 81 (28.3%) long. The facial evaluation questionnaire showed that MC had the lowest satisfaction with mean score of 2.18 ± 0.97 for overall impression and 2.15 ± 1.04 for facial parts, compared to MM and MI, with mean score of 1.80 ± 0.97 and 1.64 ± 0.74 respectively for overall impression; 1.75 ± 0.95 and 1.70 ± 0.83 respectively for facial parts. In conclusion: 1) Only 17.1% of Malaysian facial proportion conformed to the golden ratio, with majority of the population having short face (54.5%); 2) Facial index did not depend significantly on races; 3) Significant sexual dimorphism was shown among Malaysian Chinese; 4) All three races are generally satisfied with their own facial appearance; 5) No significant association was found between golden ratio and facial evaluation score among Malaysian population.

## Introduction

It is commonly said that ‘beauty lies in the eyes of the beholder’. Nonetheless, is esthetic judgment entirely subjective and purely a matter of arbitrary personal preference or could there be some scientific backings that guide and govern our perception towards beauty?

Ancient Greeks have ventured to question the meaning of beauty and believed that the world is beautiful because there is a certain order, harmony, measure and proportion between its elements [1]. For centuries, the Golden Ratio or Golden Proportion has been considered as the perfect or ideal ratio for beauty. First described by the Greek Mathematician Euclid as the extreme and mean ratio [2], whose definition reads ‘A straight line is said to have been cut in extreme and mean ratio when, as the whole line is to the greater segment, so is the greater to the lesser’; it is later discovered to have a numerical value of precisely 1 to 1.61803399 by Filius Bonacci [3,4] and it is not until the 20th century when the term “Phi” and its symbol Φ were coined by Mark Barr in commemoration of the Greek sculptor Phidias [5,6]. In essence, the golden ratio has captured the fascination of intellectuals from diverse fields and disciplines. Interestingly, golden ratio is also prevalent in nature, where it can be seen in the skeletons of animals and humans, in the arrangement of branches along the stems of plants, in the spirals of sea shells and in the wing dimensions and location of eye-like spots on moths. Even more intriguing is that Phi manifests throughout the human form such as the body, the face, the fingers and the teeth [7,8,9]. Since the divine proportion seems to evoke an aesthetically pleasing effect, it might have been hard-wired into our consciousness as a guide towards aesthetic judgment; as proposed by Jefferson in whose paper a universal standard for facial beauty measurement regardless of race, age, sex and other variables is shown [10]. In fact, several studies have also shown that beautiful faces have facial measurements close to the golden ratio [11,12,13,14,15,16,17].

Ricketts used a golden divider to prove that the harmonious faces of beautiful women followed golden proportions [18]. Presently, several studies on facial aesthetics have been carried out where facial analyses are done using lateral cephalograms [19,20,21], photographs [15,22,23,24,25] and by anthropometrical means with the employment of direct measurements [26,27,2] and three dimensional imaging [28,29], among others. More recent articles have also discussed angular and linear analyses of the soft-tissue profile [30,31,32,33,34] and lip morphology [35]. In addition, extensive literature on modern facial anthropometric data among North American white populations [36], North Eastern Nigerian [37], Indian Americans [38], North Maharashtrian [24], Malaysians [26], and Latvian [39] are available in this present day. Some studies have also attempted to classify facial shapes based on Golden Ratio to determine and compare the prevalence of faces conforming to or approaching the golden ratio across different ethnics and between genders [26,27].

There has been evidence supporting the idea that the objective appraisal of facial beauty is indeed possible and that faces which observe certain universal parameters, such as symmetry, the Neoclassical Canon and Golden Ratio, are deemed beautiful across different culture and ethnics [40,41,42]. However, there is currently little evidence of facial index in relation to the golden ratio in Malaysia. In contrast, other studies have suggested that beauty is multifactorial and its subjectivity is founded primarily on genetics [43], culture and environmental factors [44].

Studies have also been conducted to investigate the evaluation towards facial appearance where Japanese [45], Japanese-Brazillian [46], Indian Subcontinent [47] and Thai [48] laypersons were asked to perceive their own facial appearance and rate their satisfaction level, which has shown to vary across different demographic groups. However, up to this present day, there is no evidence to assess the evaluation and satisfaction of one’s own facial appearance and also to determine its association with the facial proportion in relation to the golden ratio among Malaysian population.

Hence, our study aims to provide a facial anthropometric and aesthetic analysis of the three main ethnic groups in Malaysia and to determine the prevalence of ideal faces which conform to the Golden Ratio by obtaining the facial indices and classifying subjects’ faces into different facial shape groups. Additionally, by determining the level of satisfaction of subjects towards their own facial appearance, we are interested to know whether a certain relationship exists between Facial Golden Ratio and subjects’ evaluation towards their own facial beauty, hence establishing that the Golden Ratio does influence facial attractiveness. It is also necessary to assess the profile preferences of Malaysian adults, as similar studies have not been done to date for the Malaysian population. The specific aims of this study are as follows:

1. To determine the normal anthropometrical measurements of facial height, facial width and facial indices among Malaysian population.
2. To identify and classify Malaysian population into different facial shapes based on the golden ratio.
3. To determine the existence of significant differences in facial measurements and indices between genders and three different races in Malaysia.
4. To study the evaluation of self-facial appearance among Malaysian population.
5. To determine the existence of significant differences in the evaluation of self-facial appearance between genders and three different races in Malaysia.
6. To investigate the association of facial proportion and its relation to the golden ratio with the evaluation of facial appearance among Malaysian population.
7. To determine the perception towards general facial aesthetic and preference of the lip and chin profiles among Malaysian population.

## Materials and Methods

All participants provide their written informed consent. This study was approved by the Ethical Committee of the Hospital Universiti Sains Malaysia (HUSM) [USM/JEPeM/1405203], which complies with the Declaration of Helsinki.

### Subjects and Sample Selection

The subjects of this study consisted of a total of 286 subjects, including 100 Malay (50 male 50 female), 100 Malaysian Chinese (50 male 50 female) and 86 Malaysian Indian (36 male 50 female) from 18 to 25 years of age. The sample comprising of students attending Universiti Sains Malaysia originate from all states in different parts of Malaysia and hence is a true representation of the whole Malaysian population aged 18–25 both in regards to the ethnicity ratio and also geographically. Subjects of mixed ethnicity, subjects with craniofacial deformity, and subjects with a history of orthodontic treatment or facial surgery were excluded from this study. Informed consents were obtained from the subjects before the study was carried out.

### Anthropometrical Facial Measurements

**Fig 1:**
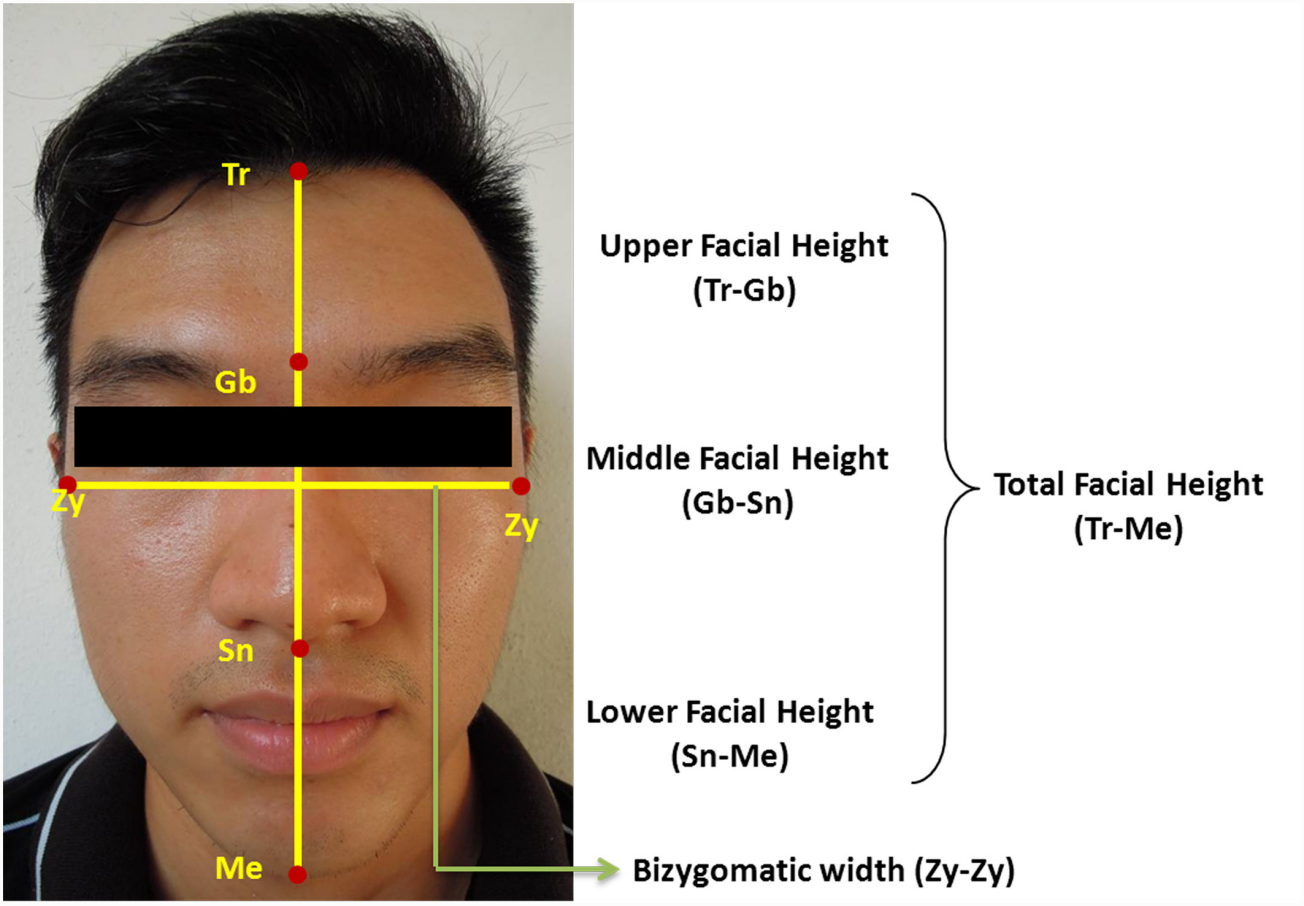
Anthropometrical landmarks and facial measurements. Tr- Trichion, Gb- Glabella, Sn- Subnasale, Me- Menton, Zy- Zygion.

Anthropometrical landmarks on the face used for facial measurements are shown in Fig 1, with their definitions provided in Table 1 [49,50]. The relevant participant has given written informed consent (as outlined in PLOS consent form) to publish the photograph in Fig 1 (Eyes were covered). Direct measuring technique, considered to be more accurate than indirect measuring technique, was employed and real-time measurement was done on the 286 subjects. The anthropometrical landmarks involved were palpated and located on the face of the subjects and a total of five measurements were taken including total facial height (Tr-Me), upper facial height (Tr-Gb), middle facial height (Gb-Sn) and lower facial height (Sn-Me) as well as width of face (Zy-Zy) using dental sliding vernier caliper (Boley Dental USA) and cephalometric protractor (Orthopli Corporation Philadelphia).

**Table 1:** Definition of anthropometrical landmarks.

Anthropometrical Landmarks	Definition
Trichion (Tr)	Anterior hairline at the midline
Glabella (Gb)	Most prominent point of the forehead on profile
Subnasale (Sn)	Junction of the inferior portion of the nasal septum and the upper lip
Menton (Me)	Most inferior soft-tissue point on chin
Zygion (Zy)	The most laterally positioned point on the zygomatic arches.

### Facial Index and Facial Shape

The ratio of the bizygomatic width (FW) to the total facial height (TFH) of each subject was calculated and recorded as the subject’s facial index (FI). Subjects were categorized into different facial shapes based on the relationship of their facial indices to the Golden Ratio (1.6–1.699 = Normal, < 1.6 = Short, > 1.699 = Long) [26,27].

### Facial Evaluation Questionnaire

Subjects were also asked to fill up a questionnaire to assess their perception towards beauty and evaluation of their own facial appearance. The questionnaire consisted of 3 parts.

Section 1 consisted of patients’ demographical data, namely name, sex, age and race.

Section 2 consisted of questions for silhouette study which included 2 series of 8 profiles each. The first series of profile showed changes in relation to the chin while the second series showed changes in relation to the lips. The average profile was No. 4. The chin or lips were protruded or retruded in 1-mm increments from the average profile, and the chin or lips positions were changed parallel to the Frankfort horizontal plane. Therefore, the chin was most protrusive in profile 1 and the most retrusive in profile 8 for the first series. Meanwhile, the lips were most protrusive in profile 8 and the most retrusive in profile 1 for the second series. Black and white silhouette profiles were used in this study to avoid any bias due to preferences in certain skin and hair style or colour. In this section, subjects were required to choose the most and least appealing profile of the lips and chin respectively. Lips and chin profiles [51] were assessed since these two facial elements are considered the most influential features of the face [29,52].

Section 3 consisted of questionnaire to assess the evaluation of the subjects towards their own facial appearance. In this section the subjects were asked to choose from 5-degree of satisfaction (1: satisfied, 2: somewhat satisfied, 3: neither satisfied nor dissatisfied, 4: somewhat dissatisfied, and 5: dissatisfied). Nine questions about the subjects’ overall impression such as the impression of the face and the outline of the face. The satisfaction score for overall impression is the mean of the responses to these nine questions. Fifteen questions about the satisfaction towards their own facial parts such as eyelids, eyes, nose, cheeks, lips, teeth, chin, lower lip, dentition, bite (anterior and posterior) and angle of jaw. The mean of the responses to these fifteen questions is recorded as the satisfaction score for facial parts.

### Statistical analysis

The data were analyzed statistically using IBM SPSS Statistics for Windows, Version 22.0 (Armonk, NY: IBM Corp. Released 2013). The measurements and results of the questionnaires were presented as mean with standard deviation (SD). The statistical significance of intergender differences in mean values of the measured parameters and satisfaction scores from the questionnaire was examined using Independent t-test while One-Way ANOVA was done to investigate the existence of statistical significance between three different races. Subjects were then categorized into different facial shapes based on the relationship of their facial indices to the golden ratio (1.6–1.699 = Normal, <1.6 = Short, > 1.699 = Long)[26,27]. Graphical representation of the subjects with different facial shapes and the frequency of the lips and chin profile preferences were obtained from Microsoft excel 2010. Associations between different variables were analyzed using Simple Linear Regression. The confidence level was set at 95% (P < 0.05).

Thirty (30) subjects were randomly selected and re-measured 1 month after the initial measurements. Intra-rater reliability was determined using the Intraclass Correlation Coefficient (ICC). Overall, the k value of the raters for each measurement was at least 0.70, indicating good intra-rater reliability. Cronbach's alpha was above 0.800 for all measurements, which indicated a high level of internal consistency.

## Results

### Intersexes disparities

**Fig 2:**
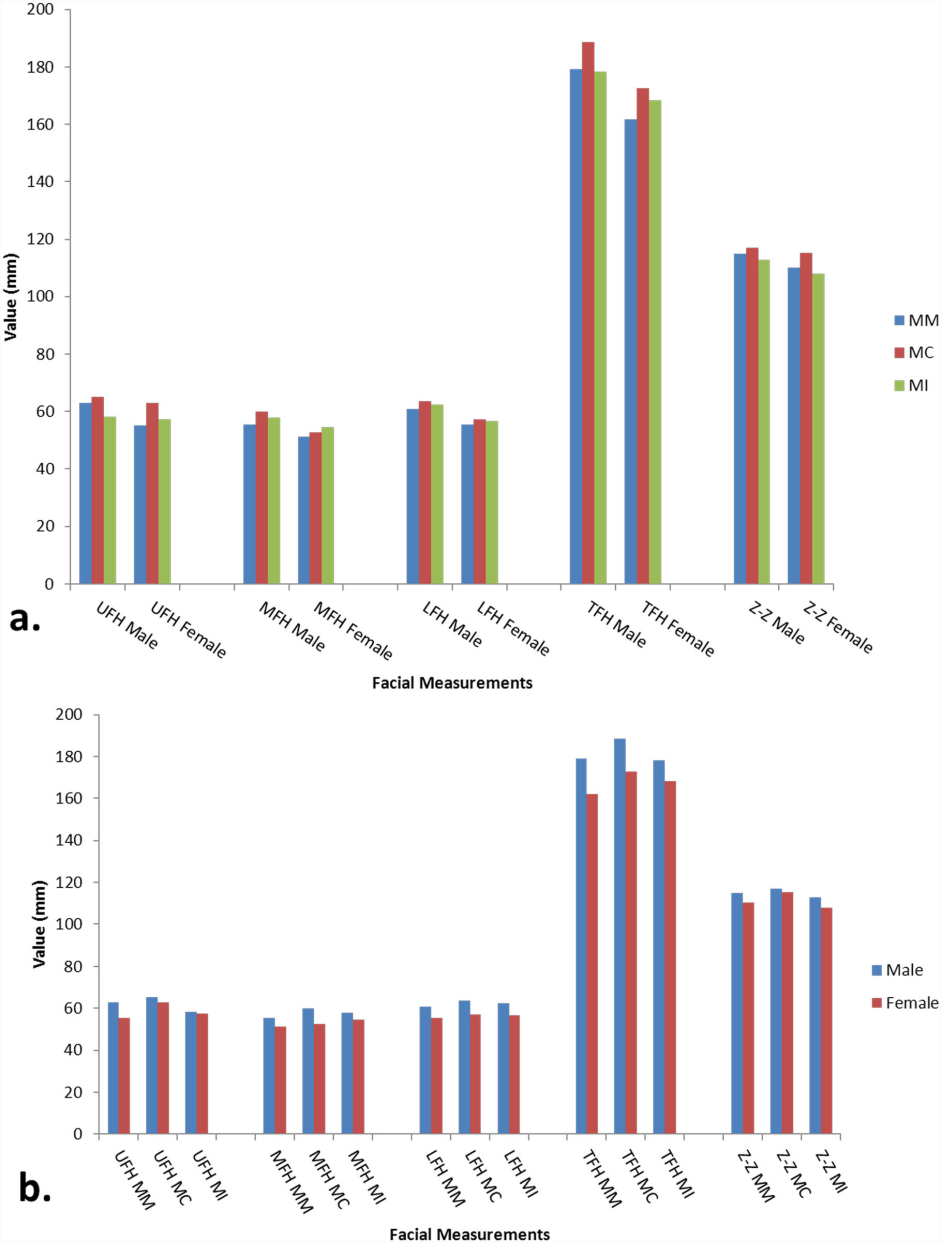
Comparison of facial measurements between, a: different genders and b: different genders in relation to different race.

Table 2, Fig 2a and 2b shows the mean facial measurements between different genders. Also illustrated is a highly significant sexual dimorphism in all three races for LFH (P< 0.001). For MFH, Malaysian Chinese (MC) (P< 0.001), Malaysian Malay (MM) and Malaysian Indian (MI) (P = 0.05) showed significant inter-gender difference. In regard to TFH, Malaysian Malay (P<0.001), Malaysian Chinese (P< 0.001) and Malaysian Indian (P = 0.001) showed significant inter-gender difference. Only MM suggested significant difference between genders for UFH (P< 0.001). For facial index, only MC showed significant inter-gender difference (P<0.05). No statistically significant difference between genders was found in all three races for facial width.

**Table 2:** Comparison of facial measurements between different genders.

Measurements	Races	Mean (SD)		P-value	95% CI	
		Male	Female		Lower	Upper
Upper facial height (UFH)	MM	62.98(7.17)	55.15(8.46)	<0.001***	5.716	10.934
	MC	65.11(7.89)	62.83(9.32)	0.190	-1.146	5.706
	MI	58.08(7.76)	57.26(6.67)	0.602	-2.286	3.922
Middle facial height (MFH)	MM	55.37(8.27)	51.26(5.65)	0.005*	1.300	6.924
	MC	59.83(5.43)	52.59(9.58)	<0.001***	4.147	10.331
	MI	57.73(5.53)	54.47(4.9)	0.005**	1.013	5.510
Lower facial height (LFH)	MM	60.70(6.69)	55.42(5.40)	<0.001***	2.877	7.700
	MC	63.49(7.02)	57.15(10.44)	<0.001***	2.807	9.867
	MI	62.44(8.11)	56.55(6.52)	<0.001***	2.750	9.033
Total Facial Height (TFH)	MM	179.05(15.27)	161.83(14.14)	<0.001***	11.386	23.070
	MC	188.43(14.00)	172.57(22.52)	<0.001***	8.414	23.298
	MI	178.25(13.17)	168.28(13.45)	0.001**	4.175	16.767
Facial Width (Z-Z)	MM	114.75(10.10)	110.12(15.15)	0.075	-0.482	9.738
	MC	117.10(11.48)	115.19(13.36)	0.445	-3.032	6.852
	MI	112.67(9.63)	107.83(13.76)	0.074	-0.472	10.145
Facial Index (FI)	MM	1.57(0.19)	1.50(0.27)	0.130	-0.021	0.162
	MC	1.62(0.18)	1.52(0.30)	0.047*	0.001	0.195
	MI	1.59(0.17)	1.58(0.20)	0.787	-0.070	0.093

(SD: Standard deviation, CI: Confidence interval, *p<0.05, **p<0.01, ***p<0.001).

### Interracial disparities

**Fig 3:**
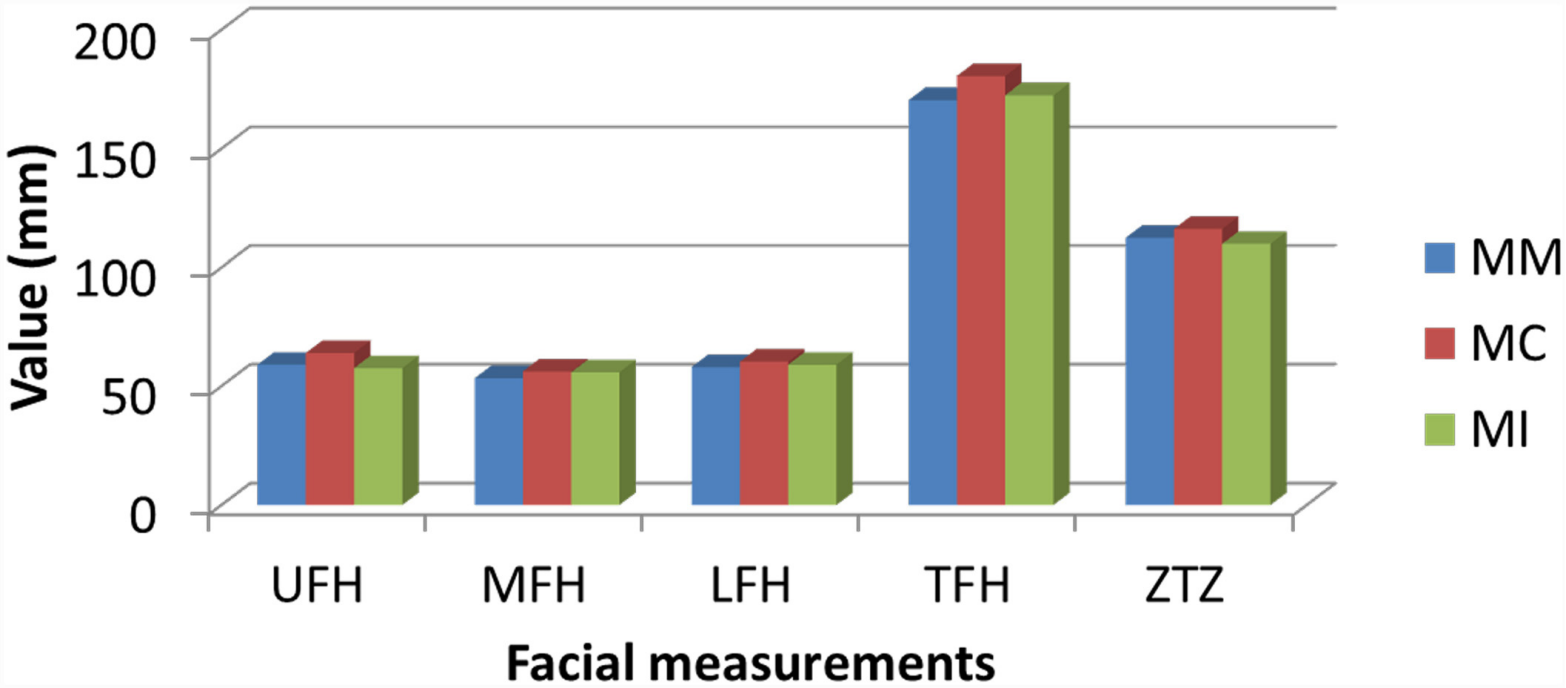
Comparison of facial measurements between different ethnics.

Table 3 and Fig 3 highlights the mean measurements of each racial group. Our findings revealed a highly significant racial difference (P<0.001) for UFH between MC and MM, and also between MC and MI. MFH was statistically significant (P<0.05) between MM and MC. In regard to TFH, statistically significant difference was shown between MM and MC (P<0.001) and between MC and MI (P<0.01). A comparison between MC and MI showed significant racial difference (P<0.01) for Zygion to Zygion. However, no significant racial difference was found in LFH measurement and facial index.

**Table 3:** Comparison of facial measurements between different ethnics.

Measurement	Mean (SD)						P-Values		
	MM		MC		MI		MM Vs MC	MM Vs MI	MC Vs MI
	Mean	SD	Mean	SD	Mean	SD			
Upper Facial Height (mm)	59.06	8.74	63.97	8.67	57.60	7.11	<0.001***	0.689	<0.001***
Mid Facial Height (mm)	53.32	7.35	56.21	8.56	55.84	5.39	0.016*	0.058	1.000
Lower Facial Height (mm)	58.06	6.60	60.32	9.40	59.02	7.76	0.142	1.000	0.812
Total Facial Height (mm)	170.44	17.01	180.50	20.29	172.46	14.15	<0.001***	1.000	0.006**
Zygion To Zygion (mm)	112.43	13.02	116.15	12.43	109.85	12.37	0.116	0.497	0.002**
Facial Index	1.54	0.23	1.57	0.25	1.59	0.19	0.771	0.435	1.000

(SD: Standard deviation, *p<0.05, **p<0.01, ***p<0.001).

Table 4 illustrates interracial difference in facial measurements among male and female subjects. Significant difference was shown between MM and MI (P<0.05) and MC and MI (P<0.001) in UFH, between MM and MC (P<0.01) in MFH as well as between MM and MC (P<0.01) and between MC and MI (P<0.01) in TFH among male subjects. Among female subjects, UFH showed significant difference between MM and MC (P<0.001) and MC and MI (P<0.01). Besides, significant difference was also shown in TFH between MM and MC (P<0.01) as well as ZTZ between MC and MI (P<0.05).

**Table 4:** Comparison of facial measurements between different races among male and female subjects.

Measurements	P-Value					
	Male			Female		
	MM Vs MC	MM Vs MI	MC Vs MI	MM Vs MC	MM Vs MI	MC Vs MI
Upper Facial Height (mm)	0.486	0.011*	<0.001***	<0.001***	0.605	0.003**
Mid Facial Height (mm)	0.003**	0.319	0.456	1.000	0.071	0.544
Lower Facial Height (mm)	0.167	0.816	1.000	0.798	1.000	1.000
Total Facial Height (mm)	0.004**	1.000	0.004**	0.006**	0.188	0.644
Zygion To Zygion (mm)	0.796	1.000	0.167	0.223	1.000	0.030*
Facial Index	0.492	1.000	1.000	1.000	0.383	0.821

(*p<0.05, **p<0.01, ***p<0.001).

### Classification of facial shapes based on the Golden Ratio

**Fig 4:**
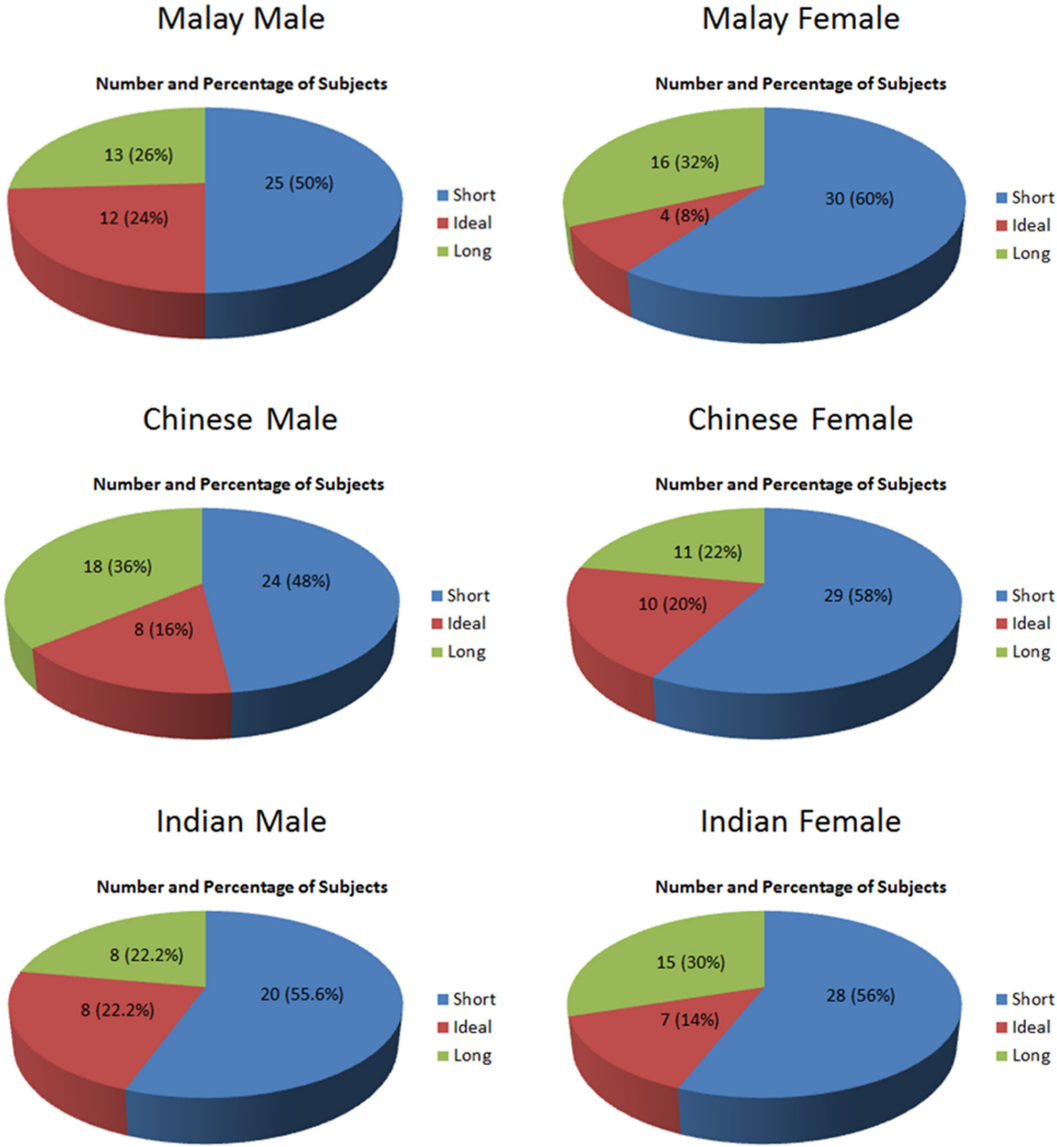
Pie charts showing facial shape classification of subjects according to the Golden Ratio.

Fig 4 revealed that in Malaysian Malay subjects, 16 (12 male, 4 female) had an ideal face, 55 (25 male, 30 female) had a short face, 29 (13 male, 16 female) had a long face. In Malaysian Chinese subjects, 18 (8 male, 10 female) had an ideal face, 53 (24 male, 29 female) had a short face, 29 (18 male, 11 female) had a long face. In Malaysian Indian subjects, 15 (8 male, 7 female) had an ideal face, 48 (20 male, 28 female) had a short face, 23 (8 male, 15 female) had a long face. Out of 286 subjects, the face shape was ideal in 49 subjects, short in 156 subjects, and long in 81 subjects.

### Mean facial evaluation score between genders and different races

Table 5 shows the mean evaluation score for both overall impression (OI) and facial parts (FP) in male and female subjects.

**Table 5:** Comparison of mean facial evaluation score between different genders.

Mean Facial evaluation Score	Mean (SD)			P-value	95% CI	
	Male	Female	Total		Lower	Upper
Mean OI	1.79 (0.72)	1.97 (0.82)	1.89 (0.77)	0.056	-0.356	0.005
Mean FP	1.84 (0.70)	1.91 (0.73)	1.87 (0.72)	0.395	-0.239	0.095

As shown in Table 6, mean evaluation scores for OI and FP were significantly higher in MC than MM and MI. Significant inter-racial difference was shown for mean OI score between MM and MC and between MC and MI (P<0.001).

**Table 6:** Comparison of mean facial evaluation score between different races.

Mean Facial Evaluation Score	Mean (SD)						P-Values		
	MM		MC		MI		MM Vs MC	MM Vs MI	MC VS MI
	Mean	SD	Mean	SD	Mean	SD			
Mean OI	1.80	0.86	2.18	0.76	1.64	0.58	0.001**	0.456	<0.001***
Mean FP	1.75	0.74	2.15	0.73	1.70	0.56	<0.001***	1.000	<0.001***

(**p<0.01, ***p<0.001).

**Fig 5:**
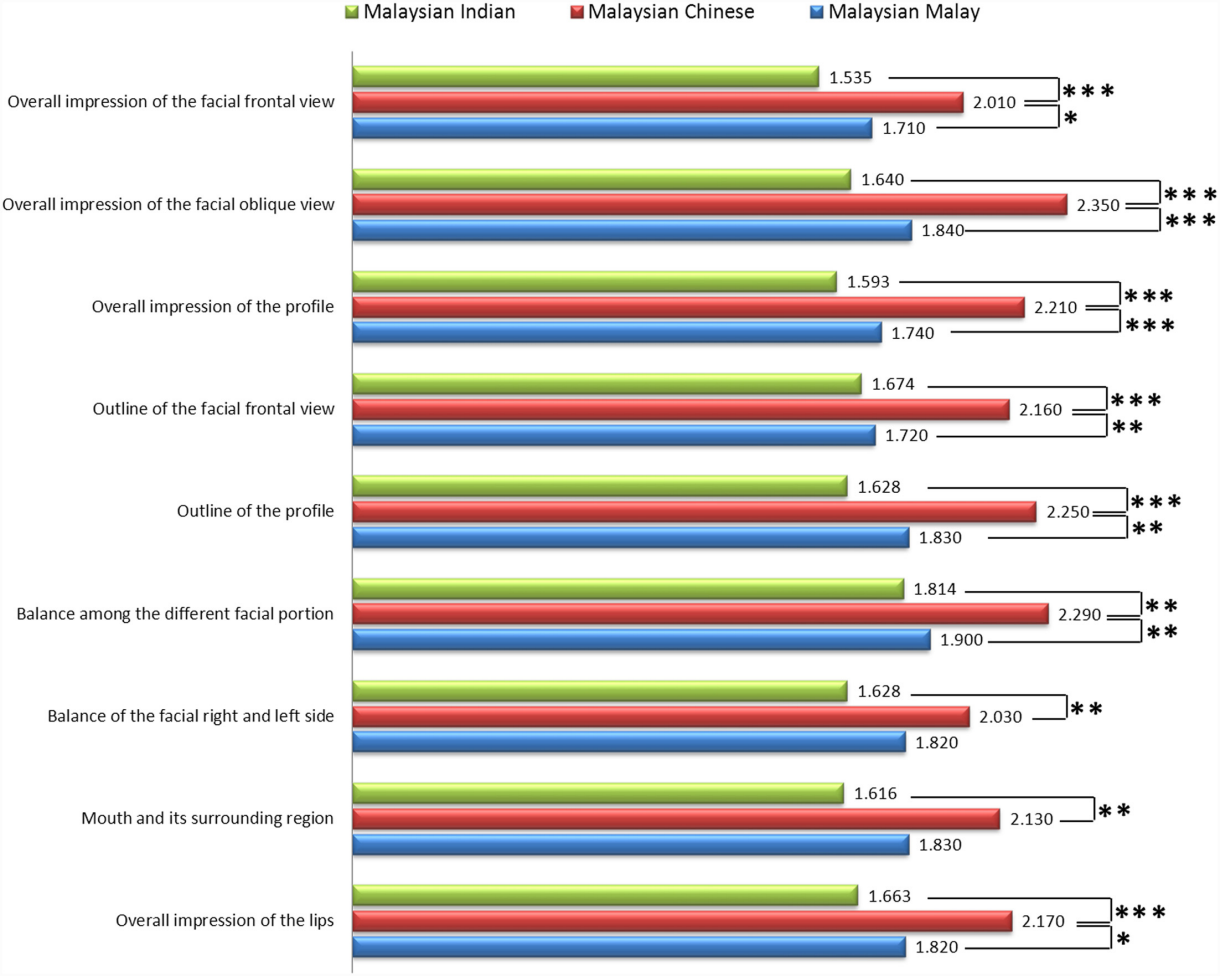
Mean evaluation score for overall impression in different races.

**Fig 6:**
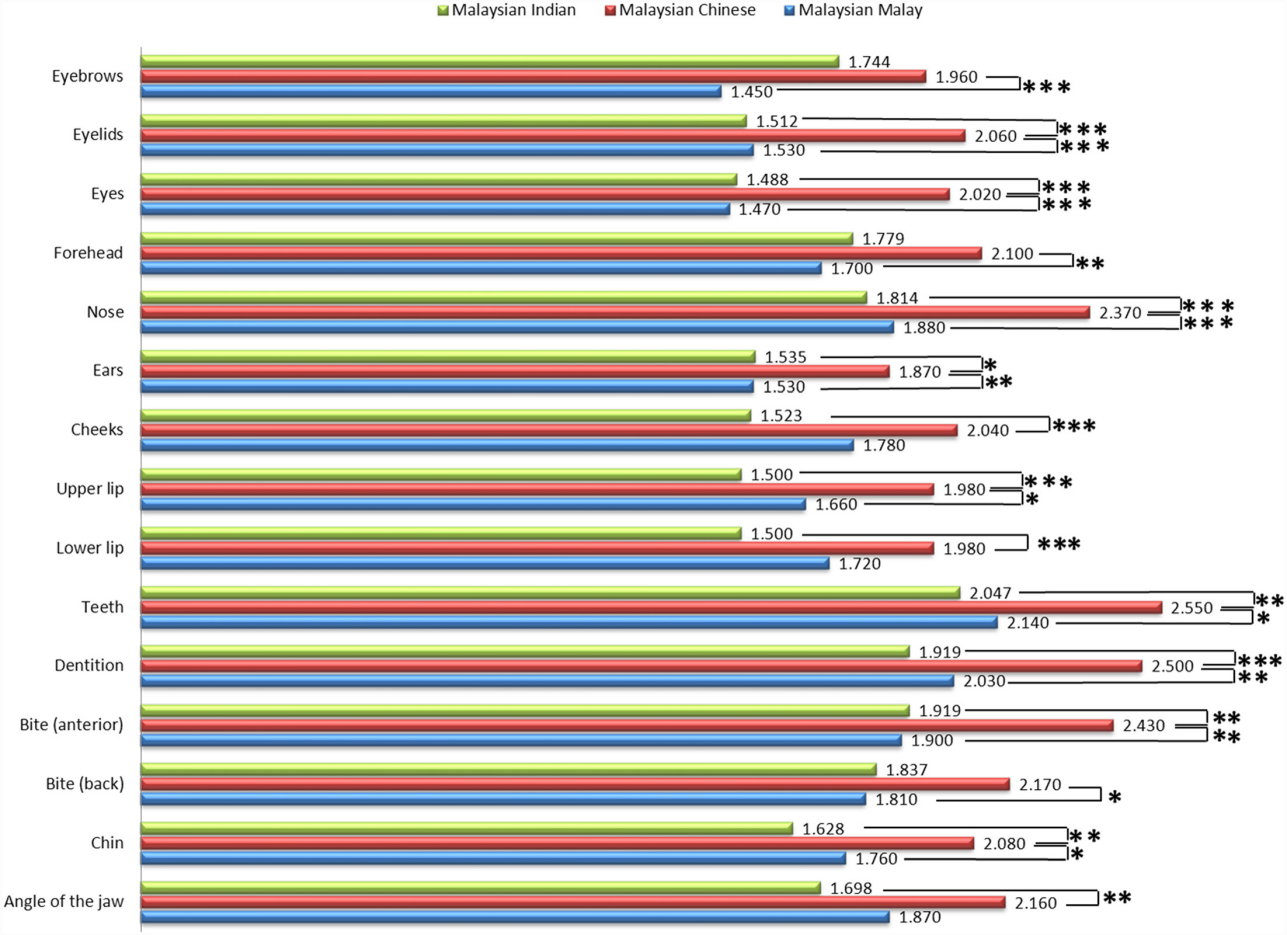
Mean evaluation score for facial parts in different races.

Figs 5 and 6 shows the mean evaluation score for each OI and FP, respectively. Significant differences between races for different variables were observed. No significant difference for mean facial evaluation score was found between different facial shapes, as shown in Table 7.

**Table 7:** Comparison of mean facial evaluation score between different facial shapes.

	P-Value		
	Ideal Vs Short	Ideal Vs Long	Short Vs Long
Mean OI	1.000	1.000	1.000
Mean FP	1.000	0.698	0.723

### Association of facial evaluation score with various factors (Sex, Race, Facial Index and Facial Shape)

Tables 8 and 9 highlight the association of mean overall impression score and mean facial parts score with various factors. No significant linear relationships are established between facial index with both mean overall impression score and mean facial parts score.

**Table 8:** Simple linear regression for association between mean overall impression score and facial index, facial shape (with ideal face group as control), sex (with male as control) and race (with Malay as control).

Variables	r2	b (95% CI)	t (df)	P-Value
Facial Index	0.002	0.152 (- 0.249, 0.553)	0.746 (284)	0.456
Facial Shape	0.003			
Short		- 0.003(- 0.254, 0.248)	-0.021 (283)	0.983
Long		0.089 (- 0.188, 0.366)	0.631 (283)	0.529
Sex	0.013			
Female		0.176 (- 0.005, 0.356)	1.917 (284)	0.056
Race	0.083			
Chinese		0.377 (0.169, 0.585)	3.567 (283)	<0.001***
Indian		-0.158 (-0.374, 0.058)	-1.436 (283)	0.152

[***p<0.001]

**Table 9:** Simple linear regression for association between mean facial parts score and facial index, facial shape (with ideal face group as control), sex (with male as control) and race (with Malay as control).

Variables	R Square	b (95% CI)	t (df)	P-Value
Facial Index	0.004	0.210 (- 0.158, 0.579)	1.122 (284)	0.263
Facial Shape	0.007			
Short		0.040 (- 0.191, 0.270)	0.339 (283)	0.735
Long		0.155 (- 0.100, 0.410)	1.196 (283)	0.233
Sex	0.003			
Female		0.072 (-0.095, 0.239)	0.853 (284)	0.395
Race	0.082			
Chinese		0.403(0.211,0.594)	4.142 (283)	<0.001***
Indian		-0.053 (-0.252, 0.146)	-0.520 (283)	0.604

[***p<0.001]

Simple Linear Regression revealed no significant linear relationships between facial shape with both mean overall impression score and mean facial parts score among subjects with long face and short face when ideal face is used as the control.

Simple Linear Regression also showed no significant linear relationships between sexes with both mean overall impression and facial parts scores. Interestingly, significant linear relationship was shown between race with both mean overall impression score (P<0.001) and mean facial parts score (P<0.001) only among MC with MM as the control. It is observed that Chinese subjects have a 37.7% higher score compared to the Malay group for mean overall impression score (CI 0.169, 0.585), and about 8.3% of variation in the mean overall impression score can be explained by race. Chinese subjects also scored 40.3% higher than the Malay subjects for mean facial parts score (CI 0.211, 0.594), with approximately 8.2% of variation in the mean facial parts score can be explained by race.

### Silhouette Studies: Lips and chin profiles preferences

**Fig 7:**
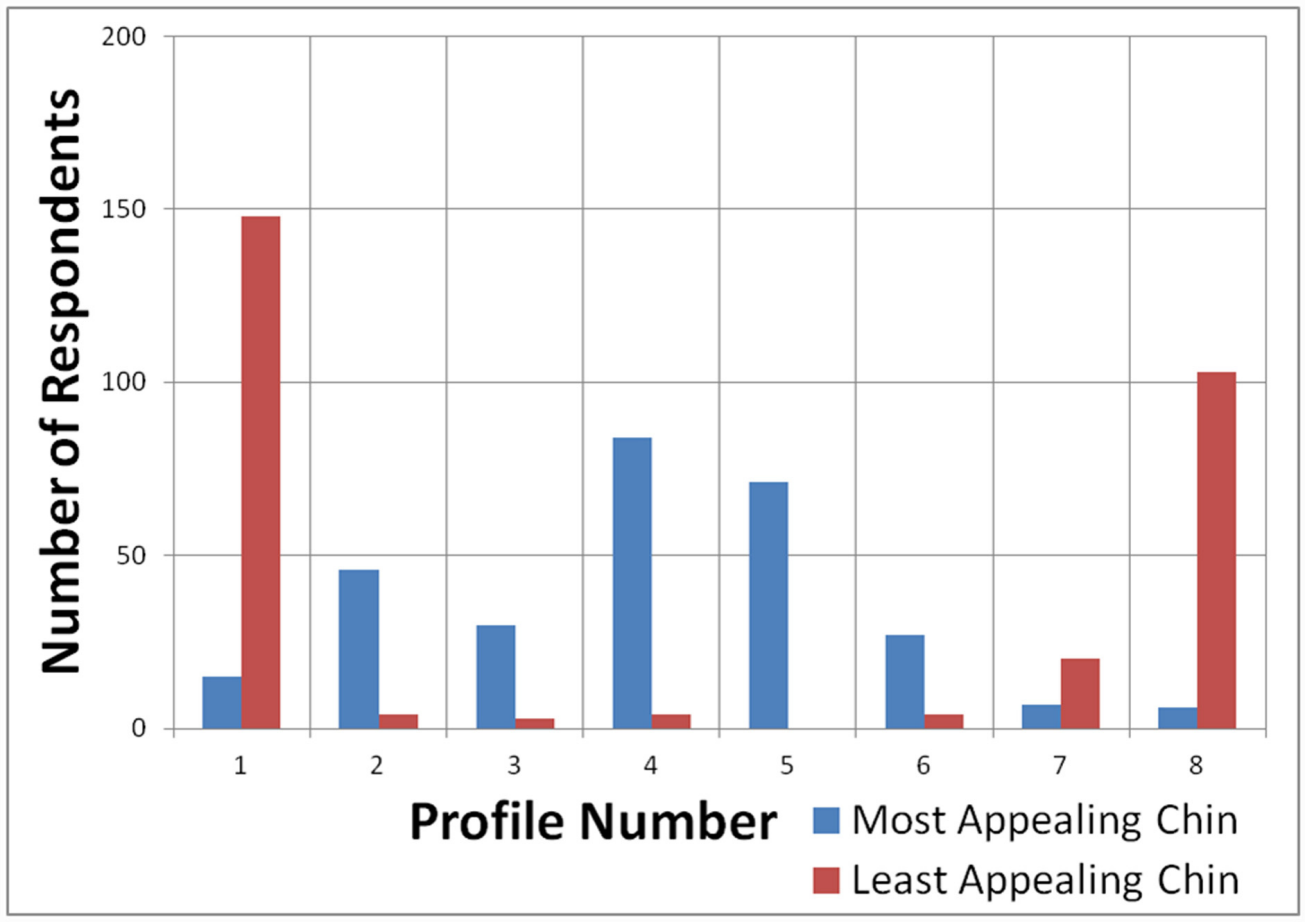
Bar chart showing frequency of chin profile preference of subjects.

**Fig 8:**
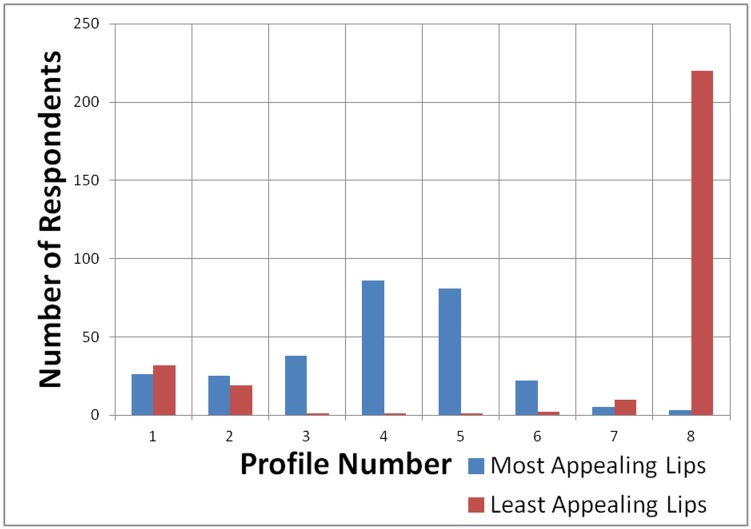
Bar chart showing frequency of lips profile preference of subjects.

Fig 7 revealed that highest percentage of subjects selected the average, orthognathic profile 4 as the most appealing for chin position and the most protrusive profile 1 as the least appealing. As shown in Fig 8, profiles 4 and 5 were chosen as the most attractive for lips position, while the most protrusive profile 8 for lips was considered the least attractive by most male and female subjects.

It is interesting to note that no subjects chose profile 5 as the least appealing chin position, which has a 1mm increment of protrusion from the average profile 4.

## Discussion

### Facial measurements of different ethnicities and populations across the globe

Extensive data of facial measurements including the total facial height and facial width of different ethnicities and populations globally from various studies are summarized in Table 10.

**Table 10:** Comparison of facial measurements in present study with studies done across the globe on different populations.

Author	Year	Sample size		Population	Methods	Total Facial Height		Facial Width	
		M	F			M	F	M	F
Farkas et al [36]	2005	30	30	North American	Spreading and sliding caliper	187.5±16.2	172.5 ±15.0	137.1±8.6	129.9±10.6
		30	30	Azerbajian		185.1±18.0	175.4 ±13.6	147.5±10.8	138.7±10.4
		30	30	Bulgarian		184.3±17.4	170.5 ±13.6	139.5±11.2	130.9±8.8
		30	30	Czech		181.7±15.8	182.9 ±16.2	134.9±26.6	126.4±28.8
		30	30	Croatian		180.1±21.2	172.6 ±17.4	140.7±12.0	133.2±13.6
		30	30	German		182.2±22.2	170.9 ±14.4	133.2±15.0	123.4±18.4
		30	30	Greek		178.7±25.8	173.8±13.8	128.6±22.8	132.2±9.6
		30	30	Hungarian		181.3±28.4	169.4±15.4	142.1±10.6	131.3±7.0
		30	30	Italian		186.0±21.2	171.4 ±18.4	143.2±11.8	133.3±8.2
		30	30	Polish		181.9±16.4	172.1 ±17.8	142.6±9.4	135.5±11.0
		30	30	Portugese		190.7±14.2	177.4 ±19.0	125.1±10.8	120.4±10.8
		30	30	Russian		184.4±16.2	174.4 ±17.4	141.2±8.8	132.3±9.6
		30	30	Slovak		183.7±17.6	169.7 ±17.5	134.7±11.0	125.0 ±11.4
		30	30	Slovenian		181.3±20.6	170.4 ±30.2	136.2±11.6	129.5±10.4
		30	30	Iranian		180.3±20.4	175.9 ±15.0	138.4±11.4	131.7±13.4
		30	30	Turkish		186.5±12.8	179.2 ±18.8	140.4±16.4	134.5±8.6
		30	30	Egyptian		176.9 ±26.8	161.4 ±17.8	139.8 ±13.8	130.3 ±10.4
		30	30	Indian		161.3±4.6	163.0 ±16.6	135.8±8.6	124.9±16.9
		30	30	Singapore Chinese		187.3±14.4	176.2 ±16.6	144.6±11.2	136.2±8.0
		30	30	Vietnamese		180.9±16.6	171.1 ±14.2	144.0±8.8	134.3±5.8
		30	30	Thai		185.1±15.4	172.8 ±17.4	147.1±11.0	138.3±12.6
		30	30	Japanese		191.4±16.6	182.8 ±14.4	147.2±11.2	141.2±11.8
		30	30	Angolan		182.6±18.2	172.4 ±17.8	139.8±10.2	132.8±8.4
		30	30	Tonga		161.8±17.0	-	133.3±2.6	
		30	30	Zulu		209.2±20.6	179.1 ±19.8	138.5±9.2	128.4±9.6
		30	30	Afro American		194.6±21.2	180.1 ±15.0	138.7±11.2	130.5±9.6
Erika[39]	2005	39	38	Latvian	“	187.3	177.0	133.1	122.4
Omar et al [38]	2005	-	102	Indian American	Photo-graphic	-	169.4 ±13.3	-	125.9±10.1
Ngeow et al [53]	2009	50	50	Malay	Spreading and sliding caliper	-	-	132.5±7.0	140.1±4.9
Ngeow et al [54]	2009	50	50	Malaysian Indian	“	-	-	136.3±4.8	126.7 ±3.9
Raji et al [37]	2010	200	143	North Eastern Nigerian	“	-	-	115.1	111.3
Jeremic et al [55]	2013	360	340	Serbian	Spreading Caliper	-	-	129.1±8.9	120.0±6.4
Kumar et al [56]	2013	300	300	Haryanvi Bania	“	-	-	130.8±7.3	123.5±7.6
Milutinovic et al [57]	2014	-	83	Caucasian	Photographic	-	-	141.7±18.8	-
Packiri-swamy et al [26]	2012	50	50	Malaysian Chinese	Spreading and sliding caliper	192.1±9.6	186.6 ±9.9	140.1±7.4	135.2±10.8
		50	50	Malaysian Indian		182.5±11.0	172.7±10.9	130.3±8.9	124.0±6.6
		50	50	Malay		189.1±8.4	179.2 ±7.8	131.3±8.7	134.0±10.2
**Present study**	2014	50	50	Malaysian Chinese	“	188.4±14.0	172.6 ±22.5	117.1±11.5	115.2±13.4
		36	50	Malaysian Indian		178.3±13.2	168.3 ±13.5	112.7±9.6	107.8±13.8
		50	50	Malay		179.1±15.3	161.8 ±14.1	114.8±10.1	110.1±15.2

(M: Male, F: Female)

The present study and Packiriswamy et al. [26] both showed similar pattern and trend in interracial and intersexes comparison. Results from both studies showed that MC reported the highest value for both TFH and facial width in male and female subjects. It has been widely maintained that, in comparison to other races, MI males were revealed to have the lowest values for both TFH and facial width while MI females also had the lowest values for facial width. Moreover, both studies also reported that male subjects showed higher values for TFH and facial width compared to female subjects in all 3 Malaysian populations, which are consistent with values of other populations across the world reported by Raji et al. [37], Ngeow et al. [53,54], Omar et al. [38] and Erika et al. [39]. In contrast, Farkas et al. [36] reported that female subjects show higher value of TFH compared to male subjects in Czech and Indian population, while female Greek subjects show higher values for Facial Width compared to male subjects.

TFH of Malaysian Chinese males (188.4mm) showed almost similar values with that of Singapore Chinese males (187.3mm) [36], which could possibly be explained by similar ancestral origin. Similar value of TFH was also shown by Latvian male subjects (187.3mm) [39]. TFH measurements of Malaysian Chinese female (172.6mm) also coincide with the values of North American females (172.5mm) [36]. TFH of Malaysian Indian males (178.3mm) was similar to that of Greek males (178.7mm) [36]. Malaysian Malay females (161.8mm) and Egyptian females (161.4mm) [36] showed almost similar TFH measurements. Facial width of female Malay subjects (114.8mm) was shown to be close to the value of North Eastern Nigerian females (115.1mm) [38].

Craniofacial parameters from our anthropometric studies on Malaysian populations can be used to provide crucial data for anatomical and anthropological research as well as research in forensic medicine. In clinical practice, these data can serve as important guidelines and references among reconstructive and plastic surgeons, maxillofacial surgeons, orthodontists and prosthodontists, particularly in analysis of treatment outcome [55,56,57,58]. For evaluation of variations in craniofacial morphology and also to detect potential pathological abnormalities, standards of anthropometric measurements should be established for Malaysian population.

### Interracial differences of facial measurements

Significant difference was shown between MM and MC (P<0.01) for TFH in both sexes. This was in contrast to the result reported by Packiriswamy et al. [26] where significant difference of TFH was reported only among female subjects while male subjects showed significant interracial difference only for facial width and zygion to zygion.

In contrast to our study where no significant difference in TFH and Zygion to Zygion for both genders was shown between MM and MI, Packiriswamy et al. [26] reported significant difference in both TFH and zygion to zygion among female subjects and TFH among male subjects.

Between MC and MI, Packiriswamy et al. [26] reported a significant difference in TFH and zygion to zygion in both genders. However, in our study significant difference in TFH was found only among male subjects and zygion to zygion among female subjects.

Packiriswamy et al. [26] showed no significant difference in facial index in both sexes between MM and MI and also between MC and MI, which is in accordance to the results reported by our study.

### Facial shapes according to the Golden Ratio

Similar to studies done by Packiriswamy et al. [26] and Saraswathi et al. [27], the highest number of subjects had short face in our study. However, in contrast to the studies done by Packiriswamy et al. [26] and Saraswathi et al. [27], which reported the least number of subjects in the long face group, our findings showed that subjects with ideal face shape were the least in all three races. It was interesting to note that although Packiriswamy et al. [26] reported that none of the Malay female had long face, our study indicated that the percentage of Malay female with long face was the second highest percentage after Chinese male; while Chinese female recorded the lowest percentage in the long face group. On this point literature is not unanimous; this could be attributed to factors such as high hairline in the measurement involving trichion and many other ethnic variables that should be taken into account.

### Facial evaluation score of different populations

As illustrated in Table 11, the mean evaluation score for both overall impression and facial parts of our study were lower than that among Thai laypersons [48], Japanese-Brazilian female laypersons [46] and Japanese laypersons [45], suggesting a higher satisfaction among Malaysian populations; but higher when compared to Indian subcontinent laypersons [47]. It is obvious in this study that although Malaysian population is generally satisfied with their own facial appearance, MC is the least satisfied of the three races.

**Table 11:** Comparison of facial evaluation score of present study with studies on other populations.

Author	Ethnicity	Sample Size	Range of Satisfaction Score	
			Overall Impression	Facial Parts
Leonardo et al [31]	Japanese Brazilian	19	1.5 to 2.9	1.4 to 2.8
Alam et al [32]	Indian subcontinent	48	1.3 to 2.1	1.3 to 1.9
Alam et al [30]	Japanese	96	2.95 to 3.5	2.55 to 3.55
Luppanapornlap et al [33]	Thai	120	1.98 to 3.26	1.95 to 3.20
**Present study**	Malaysian Malay	100	1.71 to 1.9	1.45 to 2.14
	Malaysian Chinese	100	2.01 to 2.35	1.87 to 2.55
	Malaysian Indian	86	1.53 to 1.81	1.49 to 2.05

Regarding satisfaction for each facial element, the items which most subjects are least satisfied with were “teeth” and “dentition” for both male and female, which was similar to the findings by Luppanapornlarp et al. [48].

Similar to the study done among Thai laypersons [48], no statistically significant difference was noted between male and female for both mean satisfaction score of overall impression (P = 0.056) and facial parts (P = 0.395).

### Association of facial proportion and its relation to the Golden Ratio with the evaluation of facial appearance among Malaysian population

Previous studies have been conducted using various applied method with different examined distances and ratios and facial views as well as analysed sample. Generally, the majority of investigations reported a weak correlation between golden proportion and attractiveness [28,59]. Similarly, a study done to investigate the association between the perception of facial beauty and divine proportion found that ratios of 3D facial distances were not related to attractiveness in young, white adults, as assessed by a panel of dental professionals [6]. To our knowledge, in no case has the relationship between golden proportion and perception towards subjects’ own facial appearance via facial satisfaction score evaluation been investigated. The results of the current study show that the examined facial index and facial shape have no relationship with the mean facial evaluation score. As shown in Table 9, one-way ANOVA test shows no significant difference in the mean facial evaluation score between subjects of different facial shapes. In the present study, Simple Linear Regression failed to establish significant association between facial index and mean facial evaluation score. The mean evaluation score where subjects rated their level of satisfaction on a degree of 1 to 5 in our questionnaire serves to objectify the subjectiveness of an individual’s perception towards his or her own facial esthetic. Since results from our study showed no association between facial measurement and the mean evaluation score, which is a reflection of how satisfied or dissatisfied the subjects were of their own face, hence it can be concluded that there is no association of facial proportion and its relation to the golden ratio with the evaluation of facial appearance among Malaysian population. In other words, individuals who find themselves attractive might not necessarily have facial measurements which conform to or approach the golden ratio. This might be attributed to the various psychological factors, different cultural landscapes, social acceptance and expectations, social economic status, ethnic origins, social demographic backgrounds in addition to inherent influences that affect the personal perception or judgments towards the concept of facial esthetics.

Interestingly, Simple Linear Regression shows significant association between race and mean overall impression and facial parts score among Chinese subjects (P<0.001) with Malay as the control, suggesting that perception towards own facial appearance is different across different races.

### Silhouette studies: Lips and chin profiles preferences

Previous methods used to assess facial profile attractiveness included profile line drawings [60], facial photographs and imagings [61,62] and silhouettes [63,64,65], which was adopted by our study. The overall trend in our study demonstrated that milder degrees of chin retrusion and protrusion were selected as more attractive compared to profiles with greater degrees of deviation, though the tendency was for chin protrusion to be perceived as less attractive than retrusion. This was supported by Mantzikos et al. [66] done among Japanese population who found that a straight profile was ranked the most attractive while mandibular retrognathic and prognathic profiles had poor rankings. Naini et al. [67] showed that the greater the retrusion or prominence of the chin, the lower the rating of the perceived attractiveness. Similar to a study by Maganzini et al. [68], our study indicated either a retrognathic or a prognathic mandible were found to be the least appealing by both male and female subjects.

According to Polk et al. [64], significantly more African American male and female judges preferred more retruded jaw profiles, which in is accordance to our study. Similarly, Soh et al. [69,70] reported that profiles with protrusive mandibles were perceived to be the least attractive by dental professionals, dental students and laypersons in Singapore. A study done among Japanese adults showed that mandibular retrusion was generally more favoured than mandibular protrusion [71], but the results of this study did not provide confirmation, showing that most subjects selected the most protrusive chin position as the least appealing profile. In another study, Caucasian males also preferred mandibular protrusion more than retrusion [72]. Such discrepancies might be due to cross-cultural differences between different populations.

Similar conclusions were obtained in certain studies in the orthodontic field, where in addition to the lips profile preferences, some facial profile angles were investigated. Similar to our study, Foster [73], Lines et al. [74] and Czarnecki et al. [63] used silhouette profiles with altered lip positions, where it was reported that significantly more males preferred retruded lip profiles compared to females. Other studies done among Mexican American judges [75] and Japanese orthodontists and students [65] also showed preference towards more retruded lip profiles. Our findings suggested that labial protrusion was slightly better tolerated in females compared to males, which was in line with the study by Czarnecki et al. [63].

In Summary, we found, Malaysian population has a total facial height value of 181.95 mm, facial width of 114.84 mm and facial index of 1.59. Only 17.1% of Malaysian facial proportion conformed to the golden ratio, with majority of the population having short face (54.5%). Facial index did not depend significantly on races; significant sexual dimorphism was shown among Malaysian Chinese. All three races are generally satisfied with their own facial appearance. Significant interracial differences in facial evaluation score were shown between Malaysian Chinese with Malaysian Malay and between Malaysian Chinese and Malaysian Indian; no sexual dimorphism was shown. No significant association was found between golden ratio and facial evaluation score among Malaysian population. An average profile of the lips and chin are preferred over more retrusive or protrusive profiles among Malaysian population.
